# Treatment of Severe Caries and Molar Incisor Hypomineralization and Its Influence on Oral Health-Related Quality of Life in Children: A Comparative Study

**DOI:** 10.3390/ijerph19052983

**Published:** 2022-03-03

**Authors:** Sarra Altner, Markus Ebel, Valentin Ritschl, Tanja Stamm, Christian Hirsch, Katrin Bekes

**Affiliations:** 1 Department of Pediatric Dentistry, University Clinic of Dentistry, Medical University of Vienna, Sensengasse 2a, 1090 Vienna, Austria; sarra.altner@meduniwien.ac.at; 2 Private Pediatric Dentistry Practice ‘Leo Löwenzahn’, 51465 Bergisch Gladbach, Germany; markus-ebel@gmx.de; 3 Section for Outcomes Research, Center for Medical Statistics, Informatics, and Intelligent Systems, Medical University Vienna, 1090 Vienna, Austria; valentin.ritschl@meduniwien.ac.at (V.R.); tanja.stamm@meduniwien.ac.at (T.S.); 4 Ludwig Boltzmann Institute for Arthritis and Rehabilitation, 1090 Vienna, Austria; 5 Department of Pediatric Dentistry, University of Leipzig, Liebigstr. 12, 04103 Leipzig, Germany; christian.hirsch@medizin.uni-leipzig.de

**Keywords:** molar incisor hypomineralization, caries, oral health related quality of life, oral health, pediatric dentistry

## Abstract

Background: Treatment of oral diseases can have a long-lasting impact on a child’s life well beyond its childhood years. The purpose of this study was to compare the impact of treatment on the oral-health-related quality of life (OHRQoL) of children with severe caries and severe molar incisor hypomineralization (MIH). Methods: A total of 210 children (mean age 9 years; 49% female) with severe caries (inner third of dentin) and severe MIH (post-eruptive breakdown, crown destruction) were included in the study. Both groups were matched according to age, gender, and social status. The German version of the Child Perception Questionnaire for 8–10-year-olds (CPQ-G8–10) was used before and after treatment to analyze the impact on OHRQoL. Results: Patients with severe MIH showed a significantly higher total CPQ score (17.8 (±10.6)) before treatment compared to the caries group (13.8 (±14.3)). The mean CPQ score in all subdomains decreased significantly after therapy in the MIH group. Children with severe carious lesions had similar results except in the domain “functional limitations”, as treatment led to only minor changes (2.9 (±3.6) to 2.2 (±2.6)). Conclusions: Despite a narrower treatment spectrum, patients with severe MIH experienced a greater overall improvement in OHRQoL compared to the caries group.

## 1. Introduction

Nowadays, pediatric dentists are regularly confronted with two distinct dental diseases when treating children: caries and molar incisor hypomineralization [1,2]. The most common oral disease plaguing children all over the world is dental caries. It is a global burden affecting all age groups and segments of the human population. Currently, it is estimated that 621 million children worldwide are affected by dental caries [3]. In developed countries, such as Germany, caries remains a major health concern as shown in a recent survey with 44% of 6-year-olds having at least one carious lesion [4,5]. Although it is, in principle, a preventable and curable disease, facts such as socioeconomic status, dental hygiene, and dietary factors can increase the likelihood of carious lesions’ occurrence and severity [6].

The second big challenge in pediatric dentistry is molar incisor hypomineralization, a qualitative defect that usually affects one or more permanent molars (PFM) and incisors [7]. Compared to caries, research on MIH is still in its infancy. The condition was only introduced and recognized in 2001 by the European Academy of Pediatric Dentistry (EAPD), with a recent prevalence of 13.1% worldwide and 28.7% in Germany [4,8]. The affected teeth can show signs of post-eruptive enamel breakdown and severe hypersensitivity. Often children with severe MIH are unable to carry out daily activities, such as tooth brushing, talking, smiling, and consummation of hot or cold foods. Knowledge about its etiology is still far from settled. MIH differs from caries as the treatment options are fewer since conservative treatment methods are limited due to the defective enamel interfering with the durability of many materials used [9].

Both diseases share a similarity, as they harm the patient’s oral-health-related quality of life (OHRQoL) [10,11]. OHRQoL is a complex, multidimensional construct that captures the individual’s subjective perception of oral health, functional well-being, and emotional and social well-being [12]. Several instruments assess the oral-health-related quality of life in children at different age groups, each focusing on the different phases of child development [13]. The Children Perceptions Questionnaire (CPQ) is the most widely used instrument, as it is validated, reliable, and cross-culturally adapted [14]. Although it is already known that both oral diseases impact the oral-health-related quality of life in children, majority of the studies focus on these conditions separately and rarely compare their impact on the OHRQoL. Since caries is a thoroughly researched and well-understood disease, with treatment mainly being standardized, this lack in comparison might mislead some practitioners into thinking that treatment success with carious lesions is higher as compared to MIH-affected teeth.

This study thus aims to explore whether treatment of severe caries lesions leads to more significant improvements in the quality of life than treatment of severe MIH. It does so by assessing the OHRQoL of children aged 7–11 with severe MIH and carious lesions before and after therapy. Comparing the treatment success is especially of interest for cases with severe MIH. Unlike the treatment of mildly affected teeth, where the options are narrower, severe MIH comes with a slightly wider range of possibilities. This makes severe MIH in principle more comparable to caries and the possibility of improving OHRQoL more likely.

## 2. Materials and Methods

### 2.1. Study Design

This is a matched-pair study. This study investigated the influence of treatment on OHRQoL in children with severe MIH and caries aged 7–11 years. The participants were matched by age, gender, and social status to reduce bias.

### 2.2. Subjects and Setting

Participants were recruited from Leo Löwenzahn, a private dental clinic in Bergisch Gladbach, Germany, between November 2019 and August 2020. Every dental employee at the clinic was thoroughly briefed about the study and patient-recruitment process. A leading calibrated examiner (M.E.) was chosen to perform all clinical examinations. The examiner was trained and calibrated by using standardized methods and clinical pictures for the assessment of molar incisor hypomineralization and dental caries.

The children were diagnosed based on clinical and radiological examinations, and their teeth were evaluated under artificial light using an air/water syringe, a dental mirror, and a standardized probe.

A laser-induced fluorescence with a Kavo-DIAGNOdent pen was used for caries detection combined with visual-tactile examination. When indicated (presence or suspicion of caries), a radiograph was taken, and the judgment criteria provided by the World Health Organization (WHO) and American Dental Association (ADA) were used for classification [15]. For the diagnosis of MIH, the criteria suggested by the European Academy of Pediatric Dentistry (EAPD) [16] were used.

**Figure 1:**
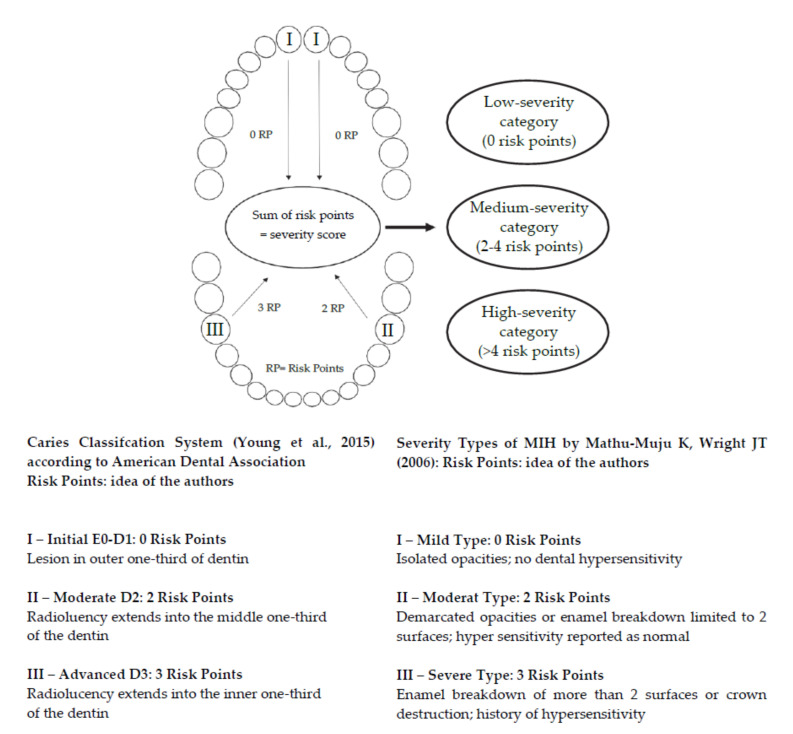
Schematic presentation for detection of the severity score of the patients’ caries-affected or MIH-affected teeth to divide them into severity categories (Michaelis et al.).

Based on the MIH severity classification by Mathu-Muju and Wright [17], a risk point system developed by Michaelis et al. [18] (Figure 1) was used to classify the patients affected by caries or MIH: (1) low-severity category (LSC; 0 risk points); (2) medium-severity category (MSC; 2–4 risk points); and (3) high-severity category (HSC; >4 risk points). According to this scheme, this study only included children aged between 7 and 11 years with high-severity carious lesions and MIH-affected teeth. This risk point system was adapted from the ADA Caries Classification [19] and categorizes the subject-related severity of caries disease similarly. It assumes that initial carious lesions (≤1/3 into dentin) and MIH severity class I [17] do not affect OHRQoL. Therefore, zero “risk points” are assigned to this category. Likewise, caries extending into the middle third of dentin is subsumed under the MIH severity class II, and two “risk points” are assigned. The maximum risk score of three points is assigned to teeth with severely advanced caries (middle third of dentin or deeper) or severity class III hypomineralized teeth (hypersensitivity and post-eruptive enamel defect). The sum of the subjects’ risk scores categorizes the patients into their corresponding severity category, making the severity of both conditions comparable. Patients with a severity score of 0 were assigned to the low-severity category (LSC), the medium-severity category (MSC) required a severity score of 2–4, and children with a severity score of >4 were included in the high-severity category (HSC).

Patients who were affected by both diseases were excluded. Furthermore, all participants who showed disease symptoms or had an illness (e.g., sinusitis, otitis media) in the last month before entering the study that could interfere with the oral findings were excluded. In addition, children with an ongoing or finished orthodontic treatment or orthodontic anomalies, such as crowding, crossbite, open bite, or any type of malocclusion in general, were also excluded. Moreover, dental anomalies, bruxism, secondary caries, enamel hypoplasia, and dental or gingival trauma were also excluded from the study.

The enrolment into this study was voluntary. The legal guardian of the patients was informed about the study verbally and signed an informed consent form after their verbal assent was obtained. This study was approved by the ethics committee of the University of Leipzig (AZ: 152/19-ek)

### 2.3. Sample Size

Sample size calculation was performed regarding the comparison between two group’s oral health-related quality of life in children with severe MIH and severe caries. A confidence interval of 95% was applied, as an error probability of 5% is quite common. A G*power analysis (www.gpower.hhu.de (accessed on 30 January 2020)) was used to compute a sample size with significant statistical power to eliminate any calculation errors. Therefore, this matched-pairs study included 210 children (103 females and 107 males). This resulted in a distribution of 105 participants for each group.

### 2.4. Variables

To assess the child’s OHRQoL before and after treatment, the validated German version of the CPQ8–10 was used [14]. This questionnaire contains 25 questions focusing on the participants’ oral health (five items), functional limitations (five items), emotional well-being (five items), and social well-being (ten items). Questions ask about the frequency of events in the child’s last four weeks. Responses are made on an ordinal scale (0 = never, 1 = once/twice, 2 = sometimes, 3 = often, 4 = every day/almost every day). Thus, the highest scores result in a less favorable OHRQoL. Only children who had full command of the German language and could complete the CPQ-G8–10 without their caregiver’s assistance were included in this study.

### 2.5. Data Sources and Measurements

The dentist and dental assistants informed all participants about the format of the present study. If a patient fulfilled all inclusion criteria, the participant and caregiver were asked about any prior diseases in the previous month that could influence OHRQoL. After ruling out any exclusion criterion, the questionnaires were distributed when written consent from the children’s caregiver was obtained. Children with more than two missing items were excluded from the study and further analysis. To ensure the patient’s anonymity, every participant’s name was replaced with a randomly assigned number.

### 2.6. Bias

As the participants were not supervised by staff while completing the questionnaire in the waiting room, it cannot be excluded that the caregivers helped answer the questionnaire in some cases even though they were instructed not to. Standardized methods were implemented, as seen in the setting section, to minimize potential sources of bias.

### 2.7. Statistical Methods

The following variables were investigated: age (in years), sex (male/female), and social status according to Winkler and Stolzenberg (low, middle, and high) [20]. Only MIH and caries-affected teeth in the high-severity category were included in this study.

Data analysis included descriptive statistics and Wilcoxon signed-rank test to determine the significance of differences in overall CPQ scores before and after therapy in each group. The scores of each domain (oral symptoms, functional limitations, emotional well-being, and social well-being) of the CPQ-G8–10 were added, and a mean value and range were calculated. Several analyses were conducted to test for significant mean differences between groups using the matched pairs approach, where similar patients were compared (according to age, sex, and social status). *p*-Values and 95% confidence intervals (CI) were calculated and considered significant if less than 0.05. For all other mean difference tests for variables, such as gender, age, and social status, the same criteria for significance were applied. A Bonferroni correction was used to compare the CPQ domains before and after therapy, with *p*-values < 0.05 considered indicative of significance.

## 3. Results

### 3.1. Participants

A total of 210 patients aged 7 to 11 years old were included in the present study (Table 1). The median age of the participants was nine years (±2) and equal in both groups, as they were matched by age. The gender was almost distributed equally with 103 females and 107 males. According to Winkler and Stolzenberg [20], more than half (54%) of the study population belonged to the middle class and 27% to the lower social class. The majority of the children were Caucasian (83%), and only 17% had a non-Caucasian background. Most patients (84%) were treated in a conscious state, followed by 11% of children treated under general anesthesia and only 5% with the help of nitrous oxide.

### 3.2. Main Results

A total of 914 teeth were diagnosed, of which 339 teeth were affected by MIH and 575 by dental caries. In the MIH group, 313 permanent teeth and 26 deciduous teeth were included. Regarding the permanent teeth, 57% were treated invasively, and 20% underwent a minimally invasive treatment. The majority of the teeth in the caries group were deciduous posterior teeth (*n* = 525; 91%). In addition, 45% of affected deciduous teeth required a filling, and 26% needed to be extracted. (Appendix A).

Table 2 shows the classification of all included teeth according to the dentition type and localization. Generally, posterior teeth were more often affected than anterior in the MIH group, and no deciduous anterior teeth were diagnosed with MIH. Most permanent posterior teeth (57%) and almost all (97%) anterior teeth required invasive therapy (Table A1).

In Table 3, CPQ results before and after therapy in the MIH and caries group are listed. The total CPQ in the MIH group was the highest, with an initial value of 17.8 (±10.6), whereas children in the caries group started with 13.8 (±14.3). The total CPQ and most subdomains decreased significantly (*p* < 0.001) after therapy in both groups (Appendix B). Compared to all subdomains, the most impact in both diseases manifested mainly in the domain “oral symptoms”. Here, the score before (6.4 (±2.9); 6.5 (±4.3)) and after (3.2 (±1.7); 3.2 (±2.3)) therapy was similar in both groups, whereas there is a significant difference (*p* > 0.01) in the subscale “functional limitations” before treatment since the MIH group had a higher score (4.9 (±3.3)) than the caries group (2.9 (±3.6)) to begin with. However, after therapy, both groups achieved similar results (2.1 (±1.9) and 2.2 (±2.6)) (Table A2). The total CPQ was higher in the MIH group with 17.8 (±10.6) than the caries group (13.9 (±14.2)), which demonstrates a significant difference of sum scores (*p* < 0.02). After therapy, a similar total CPQ sum was achieved by both groups (7.5 (±4.7); and 7.3 (±6.7)) although, yet again, the MIH group had a slightly higher score.

The CPQ questionnaire also included two introductory questions on the child’s overall well-being and oral health. The distribution of the ratings for these questions was examined individually for each group (Table 4). The “overall well-being” in the MIH group before therapy was mostly positively rated with 79% “very good” ratings, whereas there were more mediocre or negative ratings in the caries group. After therapy, the “excellent” ratings in the MIH group almost doubled (15% to 29%). Before therapy, children with MIH had a compromised oral well-being, as 63% opted for the answer “moderate”. Children with severe carious lesions had mixed feelings since the votes for the ratings are somewhat equally distributed.

Table 5 portrays the difference in means of the CPQ domains between the MIH and caries groups. The means of total CPQ and domains “functional limitations” and “emotional well-being” were higher in the MIH group and thus led to a significance compared with the caries group.

## 4. Discussion

The overall results showed that children with severe forms of MIH and carious teeth had a significantly better OHRQoL score after treatment. Interestingly, the overall average increase in OHRQoL was greater for children with severe MIH compared to those with severe caries. This refuted our initial assumption that the broader therapy spectrum and more established best-practice standards for caries result in a greater increase in overall OHRQoL after treatment. The lower original total CPQ sum can partially explain the greater improvement of the total CPQ score for the MIH group before treatment. However, after therapy, the total CPQ score in both groups was significantly lower and at a similar level, implying success regarding the well-being in both groups. These results align with previous studies that have focused on the diseases MIH and caries separately. For example, Dias et al. [21] showed that school children with severe MIH have a reduced OHRQoL with a total CPQ of 15.1 (±10.9), which is comparable to ours (17.9 (±10.6)). A recent study in Brazil [22] also arrived at similar findings regarding OHRQoL in caries-affected children. Children with untreated caries had a mean score of 15.2 (±12.7), which is similar to our findings before therapy (13.9 (±14.2)). However, to the best of our knowledge, this is the first investigation in the field to compare the treatment success of children with severe MIH to those with severe carious lesions.

In the present study, in the caries group, only a few affected permanent teeth needed treatment compared to the MIH group. Severe caries in these teeth are unlikely due to the age group of the participants, as the progression of caries takes more time with newly erupted teeth. The possibility of them already being severely damaged was thus low. In comparison, the MIH group consisted mostly of permanent teeth, as the probability and occurrence for deciduous molar hypomineralization (DMH) is known to be rather rare in deciduous teeth (9%) [23].

Our findings reflect the difference in treatment options for MIH and caries. Children with carious lesions in the primary dentition were not solely treated with direct restoration, as sometimes an endodontic treatment or extraction was required. The MIH-affected teeth were mostly treated with fillings and preventive measurements, such as fissure sealants. Only one tooth was extracted in this group.

Although both groups showed surprisingly similar overall scores at the last elicitation of the OHRQoL scores, indicating comparable satisfaction with the treatment, there were substantial differences in the sub-domains. Before and even after therapy, the domain “oral symptoms” were similar in both groups, which was to be expected. Due to constant penetration of oral bacteria through the hypomineralized and often defective enamel, inflammatory reactions in the pulp can cause hypersensitivity that is characteristic of MIH-affected teeth. Caries can have a similar effect on the pulp as with progressive penetration of bacteria, namely a pulp inflammation, the occurrence of which leads to severe pain.

In the domain “functional limitations”, the MIH group stood out with a significantly higher score before therapy compared to caries, which means that children with severely affected MIH teeth have more limitations, oftentimes because they suffer more than children with carious lesions. This shows that untreated MIH teeth lead to serious restrictions in the masticatory process, which is eased by treatment to a greater degree than in those with severe caries. Multiple studies corroborate these findings and show similar results for the functional limitations (4.5 (±1.6) and 4.2 (±4.2)) [14,24]. One explanation why the functional limitations are not as comprehensive for carious teeth is the phenomenon of chronification. Due to the formation of tertiary dentine, a protective shield to avoid further pulp irritation is built. This means that even a tooth with an advanced lesion and large cavity can be asymptomatic for a certain amount of time.

The domain “emotional well-being” also shows a significantly higher value in the MIH group before therapy compared to the caries group, meaning that the patients experienced more emotional distress. Dias et al. observed similar results in the emotional well-being domain with 6.5 (±6.5) to ours (5.1 (±4.0)). In children suffering from MIH, the molars are extensively affected and typically also the buccal surface of the incisor. This is connected with an impairment in oral aesthetics, which might dissuade children from smiling and indirectly affect the parents. This can have a reflexive negative influence on the emotional wellbeing of the child, which is alleviated through the restoration of adequate aesthetics. The emotional well-being in the caries group is less affected since most of the anterior teeth that are affected are deciduous teeth, and due to the age group, it is a common, socially accepted phenomenon that children do have anterior gaps for a specific time until the permanent teeth erupt. A Brazilian study [22] that investigated the impact of caries on the OHRQoL had similar results in the emotional well-being section (3.5 (±4.2)) as ours (2.8 (±3.9)) as well as a study from Turkey [25] that achieved an almost identical value after treatment of carious lesions.

Before therapy, the domain “social well-being” was similar in both groups, yet patients with MIH show a significantly better OHRQoL than the caries group after therapy. One of the reasons for the general positive effect in both groups is that malocclusion and aesthetic limitations have similar effects on social as they do on emotional well-being, which becomes especially important in this age group. Good results after therapy typically lessen the stress due to negative social feedback.

MIH fares better because treatment of carious lesions includes extractions and usage of stainless-steel crowns, which is in itself not as aesthetically pleasing as a filling and therefore does not improve social well-being as well as in the MIH group. Similarly, 43% of anterior teeth were removed in the caries group, which may not be an aesthetically ideal solution.

Furthermore, many studies have shown that socioeconomic status significantly impacts caries occurrence in children [6]. In the caries group, almost a quarter of all children included were non-Caucasian, whereas in the MIH group, it was only one-tenth. This is in accordance with findings that show that socioeconomic factors can make it more likely to incur caries and can themselves have an impact via spillover effects on the OHRQoL [26]. Several studies show that MIH seems to be independent of socioeconomic influence and other factors that negatively affect the OHRQoL [27,28,29]. Furthermore, untreated caries can be associated with infections, thereby indirectly leading to discomfort, pain, lack of appetite, and weight loss. Facts that might similarly depress the well-being of the patients.

A particular strength of this study is filling an important gap in the literature regarding the comparison between severe caries and severe MIH that might prove especially interesting and relevant for practitioners and their expectations regarding treatment success. The results should encourage general dentists to treat severely affected MIH teeth and carious lesions according to guidelines, as they lead to a significant betterment of OHRQoL.

A potential weakness of this study is the challenges of comparing two different diseases. Differences in etiology, therapy options, and causes that effect the patients’ OHRQoL can impact the recovery potential and process and the associated OHRQoL. One should bear this in mind when interpreting the results. Although existent, these risks are attenuated in our case since we restricted our focus to cases of severe MIH and caries, which have a, relatively speaking, similar treatment spectrum. We also restrict the evaluation period to a one-year follow-up, which reduces the effect that different etiological factors have on the treatment success, as socioeconomic factors might impact the treatment success over the long run and thereby influence the OHRQoL.

## 5. Conclusions

We observed a great improvement in OHRQoL in patients with severe MIH and severe caries. Despite a slightly more restricted spectrum of therapy options, there was a greater relative improvement in the MIH group. This can partly be explained by the lower level of OHRQoL before treatment in the MIH group and more aesthetically optimal treatment solutions for MIH patients.

## Figures and Tables

**Table 1:** Summary of Sample Data (*n* = 210 patients).

	All *n* (%)	MIH *n* (%)	Caries *n* (%)
Gender			
Male ♂	107 (51)	52 (47.6)	55 (52.4)
Female ♀	103 (49)	53 (52.4)	50 (47.6)
Social status *			
Low	57 (27.1)	9 (8.6)	48 (45.7)
Medium	114 (54.3)	68 (64.8)	46 (43.8)
High	39 (18.6)	28 (26.7)	11 (10.5)
Ethnicity			
Caucasian	174 (82.9)	95 (90.5)	79 (75.2)
Non-Caucasian	36 (17.2)	10 (9.5)	26 (24.8)
Age **			
Mean	9.0 years	9.0 years	9.0 years
7 years	12 (5.7)	6 (5.7)	6 (5.7)
8 years	66 (31.4)	32 (30.5)	32 (30.5)
9 years	59 (28.1)	30 (28.6)	30 (28.6)
10 years	53 (25.2)	26 (24.8)	26 (24.8)
11 years	20 (9.5)	10 (10.5)	10 (10.5)
Treatment methods			
Conscious	177 (84.3)	96 (91.4)	81 (77.1)
Nitrous oxide	11 (5.2)	5 (4.8)	6 (5.7)
General anesthesia	22 (10.5)	4 (3.8)	18 (17.1)

* after Winkler and Stolzenberg; ** at the beginning of treatment.

**Table 2:** Affected and Treated Teeth in MIH and Caries Group.

	MIH Teeth (*n* = 339) *n* (%)				Carious Teeth (*n* = 575) *n* (%)			
	Anterior Teeth 46 (13.5)		Posterior Teeth 293 (86.4)		Anterior Teeth 74 (12.8)		Posterior Teeth 501 (87.1)	
Therapy Index	Deciduous 0 (0.0)	Permanent 46 (100.0)	Deciduous 26 (8.8)	Permanent 267 (91.1)	Deciduous 56 (75.6)	Permanent 18 (24.3)	Deciduous 469 (93.6)	Permanent 32 (6.3)
No therapy	0 (0.0)	2 (0.9)	0 (0.0)	1 (0.4)	9 (16.1)	0 (0.0)	6 (1.3)	0 (0.0)
Non-invasive	0 (0.0)	0 (0.0)	2 (7.7)	33 (12.4)	1 (1.8)	0 (0.0)	18 (3.8)	1 (3.1)
Minimal-invasive	0 (0.0)	0 (0.0)	5 (19.2)	67 (25.1)	0 (0.0)	5 (27.8)	26 (5.5)	17 (53.1)
Invasive	0 (0.0)	44 (95.6)	16 (61.5)	153 (57.3)	22 (39.3)	13 (72.2)	239 (51.0)	14 (43.8)
Endodontic treatment	0 (0.0)	0 (0.0)	0 (0.0)	1 (0.4)	0 (0.0)	0 (0.0)	9 (1.9)	0 (0.0)
Prosthodontic treatment	0 (0.0)	0 (0.0)	1 (3.8)	11 (4.1)	0 (0.0)	0 (0.0)	0 (0.0)	0 (0.0)
Endodontic + prosthodontic treatment	0 (0.0)	0 (0.0)	0 (0.0)	0 (0.0)	0 (0.0)	0 (0.0)	48 (10.2)	0 (0.0)
Extraction	0 (0.0)	0 (0.0)	2 (7.7)	1 (0.4)	24 (42.9)	0 (0.0)	123 (26.2)	0 (0.0)

**Table 3:** Comparison of the Means and Confidence Intervals for CPQ Subscale Scores for the MIH and Caries Group Before and After Therapy.

	Before							After						
	MIH			Caries				MIH			Caries			
CPQ Domain	Mean CPQ	CI 95%		Mean CPQ	CI 95%		*p*-Value	Mean CPQ	CI 95%		Mean CPQ	Cl 95%		*p*-Value
		Lower	Upper		Lower	Upper			Lower	Upper		Lower	Upper	
Total CPQ	17.88 (10.60)	10	25	13.88 (14.23)	4	19	0.022	7.54 (4.75)	4	10	7.30 (6.71)	2	12	0.767
Oral symptoms	6.36 (2.99)	4	8	6.54 (4.30)	3	9	0.724	3.16 (1.70)	2	4	3.22 (2.36)	1	5	0.841
Functional limitations	4.90 (3.33)	2	7	2.97 (3.64)	0	5	<0.001	2.08 (1.90)	0	3	2.22 (2.63)	0	4	0.652
Emotional wellbeing	5.06 (4.01)	2	8	2.83 (3.89)	1	4	<0.001	1.97 (2.19)	1	2	1.21 (1.66)	0	2	0.005
Social wellbeing	1.55 (2.60)	0	3	1.53 (3.46)	0	2	0.964	0.33 (0.79)	0	0	0.66 (1.47)	0	1	0.049

**Table 4:** Comparison of Questions concerning Overall well-being and Oral Health (*n* = 210 patients) Before and After Therapy.

	MIH (*n* = 105)					Caries (*n* = 105)			MIH Vs. Caries	
	Overall Well-Being *n* (%)		Oral Health *n* (%)		Overall Well-Being *n* (%)		Oral Health *n* (%)		Overall Well-Being	Oral Health
	Before Therapy	After Therapy	Before Therapy	After Therapy	Before Therapy	After Therapy	Before Therapy	After Therapy	*p*-Value	
Excellent	16 (15.2)	30 (28.6)	1 (1.0)	3 (2.9)	18 (17.1)	19 (18.1)	0.727	4 (3.8)	0.727	1
Very good	83 (79.0)	63 (60.0)	10 (9.5)	22 (21.0)	54 (51.4)	60 (57.1)	1	36 (34.3)	1	0.307
Good	6 (5.7)	12 (11.4)	17 (16.2)	56 (53.3)	24 (22.9)	24 (22.9)	0.280	28 (26.7)	0.280	0.001
Moderate	0 (0.0)	0 (0.0)	66 (62.9)	24 (22.9)	8 (7.6)	1 (1.0)	1	32 (30.5)	1	1
Poor	0 (0.0)	0 (0.0)	11 (10.5)	0 (0.0)	1 (1.0)	1 (1.0)	1	5 (4.8)	1	0.236

**Table 5:** Difference in Means between MIH and Caries group in CPQ domains.

CPQ Domain	MIH	Caries	*p*-Value
Oral symptoms	3.20 (2.64)	3.32 (3.34)	0.766
Functional limitations	2.83 (2.83)	0.75 (2.57)	<0.001
Emotional well-being	3.09 (3.81)	1.62 (2.82)	0.002
Social well-being	1.22 (2.58)	0.88 (2.56)	0.335
Total CPQ	10.33 (9.35)	6.57 (9.42)	0.004

## Data Availability

The datasets of this article are available from the corresponding author on a reasonable request.

## Appendix A

**Table A1:** Overview of the Therapy Score and Treated Teeth in the MIH and Caries Group.

	MIH Teeth (*n* = 340) *n* (%)		Carious Teeth (*n* = 575) *n* (%)	
Therapy Index	Deciduous Teeth 26 (7.5)	Permanent Teeth 314 (92.3)	Deciduous Teeth 525 (91.3)	Permanent Teeth 50 (8.7)
No therapy	0 (0.0)	4 (1.2)	15 (2.6)	0 (0.0)
Non-invasive	2 (0.5)	33 (9.7)	19 (3.3)	1 (0.2)
Minimal-invasive	5 (1.4)	67 (19.7)	26 (4.5)	22 (3.8)
Invasive	16 (4.7)	197 (57.8)	261 (45.4)	27 (4.7)
Endodontic treatment	0 (0.0)	1 (0.3)	9 (1.6)	0 (0.0)
Prosthodontic treatment	1 (0.3)	11	0 (0.0)	0 (0.0)
Endodontic + prosthodontic treatment	0 (0.0)	0 (0.0)	48 (8.4)	0 (0.0)
Extraction	2 (0.6)	1 (0.3)	147 (25.6)	0 (0.0)

## Appendix B

**Table A2:** Means and Confidence Intervals for CPQ Subscale Scores for the MIH and Caries Group Before and After Therapy.

	MIH							Caries						
	Before			After				Before			After			
CPQ Domain	Mean CPQ	CI 95%		Mean CPQ	CI 95%		*p*-Value	Mean CPQ	CI 95%		Mean CPQ	Cl 95%		*p*-Value
		Lower	Upper		Lower	Upper			Lower	Upper		Lower	Upper	
Total CPQ	17.88 (10.60)	10	25	7.54 (4.75)	4	10	<0.001	13.88 (14.23)	4	19	7.30 (6.71)	2	12	<0.001
Oral symptoms	6.36 (2.99)	4	8	3.16 (1.70)	2	4	<0.001	6.54 (4.30)	3	9	3.22 (2.36)	1	5	<0.001
Functional limitations	4.90 (3.33)	2	7	2.08 (1.90)	0	3	<0.001	2.97 (3.64)	0	5	2.22 (2.63)	0	4	0.087
Emotional wellbeing	5.06 (4.01)	2	8	1.97 (2.19)	1	2	<0.001	2.83 (3.89)	1	4	1.21 (1.66)	0	2	<0.001
Social wellbeing	1.55 (2.60)	0	3	0.33 (0.79)	0	0	<0.001	1.53 (3.46)	0	2	0.66 (1.47)	0	1	0.018
